# Wound Photobiomodulation Treatment Outcomes in Animal Models

**DOI:** 10.1155/2019/6320515

**Published:** 2019-07-28

**Authors:** Alondra Lopez, Cord Brundage

**Affiliations:** Animal and Veterinary Sciences, Cal Poly Pomona, Pomona 91768, USA

## Abstract

The possibilities that photobiomodulation has brought on to the medical field are ever expanding and the scope it has reached is infinite. Determining how this relatively new treatment technique can be incorporated into the veterinary medical field is of interest to many medical professionals. In this review, we examine the treatment outcomes of low-level-laser therapy (LLLT) in different animal models to pinpoint any similarities between the studies. A search was conducted to identify LLLT studies using different animal models with an open or closed wound. The studies were compared to identify the laser parameters that resulted in positive treatment outcomes. The overall result of the studies examined indicated that daily laser exposure at a wavelength of a 600 or 800 nm range was the most beneficial across the rodent studies regardless of health status or wound type. More studies on rabbit, canine, and equine models are needed to explain the inconsistent results reviewed and find the correct treatment parameters for these species. Further research involving LLLT studies that focus on different factors including health status, treatment interval, wavelength, and energy density is needed to help validate our knowledge about the efficacy of using photobiomodulation in the veterinary medical field.

## 1. Introduction

Photobiomodulation using low-level lasers is an increasingly popular treatment modality that has sparked the interests of medical professionals. Lasers are popularly used in the cosmetology and dermatology field, but in other medical fields, its efficacy and utility are still being evaluated [1]. Since the invention of the laser in the 1960s, it has shown potential as a versatile therapeutic device, yet the empirical evidence available is highly variable and limited in some species. Endre Mester, also known as the father of photobiomodulation, carried out multiple studies using LLLT in the 1970s. Mester was one of the first people to demonstrate, among other findings, that a low-level helium-neon laser prompted the healing of burn wounds in mice [2]. His research in the field of photobiomodulation pioneered the path for many others and posed many questions about the possible effects of LLLT [2]. To this day, a complete understanding of the biochemical mechanism of action and targets of low-level lasers is still the subject of ongoing research [3]. With the breadth of lasers and settings available and the early knowledge we have about the effects of these variables, it has been hard for laser therapy to be explicitly validated for specific medical conditions. Scientists have begun to evaluate the use of LLLT as an alternative or adjunct to modern day techniques used for wound treatment.

There are four different stages of wound healing: hemostatic, inflammatory, proliferative, and maturation phase [4]. Many factors can affect a wound's ability to heal properly. Some of these factors include chronic infections and underlying diseases that can cause disturbances in the healing process [5]. It is important to find the most effective mechanism for wound healing, as this will help minimize the risk of a secondary infection, prevent delayed closure, and limit tissue death. A wound is not completely healed until the tissue looks and functions anatomically normal [5]. An acute wound can heal within a reasonable amount of time or, if left untreated, it can become chronic and fail to progress through the normal phases of the healing process [5]. Determining the effect LLLT has on one or all of these phases and the progression between phases has become a topic of considerable research. It is suggested that laser biostimulation is typically most effective in the proliferative phase of wound healing [6]. LLLT also has a positive effect on the biological activity of mast cells which result in the enhancement of wound healing [7]. Identifying what mechanisms are targeted by low-level lasers and what the influence of those mechanisms are in the wound healing process can help determine the viability of laser treatment.

LLLT is believed to promote the regeneration of tissues by reducing inflammation and consequently pain [8]. This type of laser has low energy density levels that cause a photochemical reaction in the inflicted area [3]. Higher density level lasers exhibit a thermal mechanism that can be used in cutting tissue. Low-level lasers, however, result in the direct absorption of light emitted from the laser diode into the targeted tissue without producing a significant change in tissue temperature, prompting the term “cold laser” [3]. LLLT's action is present at the cellular level. Evidence suggests that the mitochondria in exposed cells absorb photons emitted from the laser, which then stimulates increased ATP production and lowers levels of reactive oxygen species [9]. This results in the activation of certain transcription factors that give off gene transcript products that cause the beneficial effects of LLLT [8]. The efficacy of low-level lasers has been investigated at the* in vitro *level with* in vivo* animal studies and in human clinical trials [10]. Results indicate that for a correct treatment protocol differing laser parameters must be taken into account. Laser class, wavelength, power, type of beam, duration of exposure, and the type of tissue that is being exposed are all critical variables in the effectiveness of LLLT. Comparing the results between different species, e.g., a canine versus a rodent model, can help identify the discrepancies and similarities between them pinpointing the effects of the differing variables. In this literature review we will be examining the reported effects of LLLT in relation to species, evaluating the different variables including patient health status and laser parameters that can influence open versus closed wound healing.

## 2. Rodents

### 2.1. Mouse

Mice are the most commonly used species in research. A number of studies have evaluated the effects of LLLT for open and closed wounds in this species (see Table 1). Two studies evaluating laser parameters in open wound treatments in mice were conducted by Demidova-Rice et al. in 2007 [1] and Gupta, Dai, and Hamblin in 2014 [11]. Demidova-Rice et al. used mice (n = 139) of three different strains inflicted with 10 × 13 mm dorsal open wounds [1]. Mice were grouped into specific categories based on energy densities with their corresponding wavelengths (nm). The groups were split as follows: 1, 2, 10, and 50 J/cm^2^ (635±15 nm); 1 and 2 J/cm^2^ (632.8 nm); and 1 J/cm^2^ (670, 720, and 820 nm ± 15) [1]. Laser treatment occurred as a single exposure 30 minutes after wound infliction. Wound areas were compared daily between treatment groups. The 635 nm (2 J/cm^2^) and the 820 nm (1 J/cm^2^) wavelength groups had a positive effect on wound contraction. Gupta et al. inflicted mice (n = 30) with a 1.2 × 1.2 cm open wound on the dorsal thorax [11]. The mice were divided into groups treated with a 635±15, 730, 810, or 980 nm wavelength with a set energy density of 4 J/cm^2^ [11]. The laser-treated groups were given a single laser exposure 30 minutes after wound infliction and once daily for seven consecutive days after [11]. Both studies showed a greater reduction in wound area in the laser-treated groups using an 800 nm wavelength range. Gupta and colleagues also evaluated histopathological factors which included the production of collagen, cellular proliferation, and complete reepithelialization in which the 635 and 810 nm groups using an energy density of 4 J/cm^2^ exhibited an enhanced effect from LLLT, compared to the other two groups [11]. Both studies found LLLT to have a positive effect when using a wavelength in the 800 nm range, yet the energy densities and treatment frequency were different. Evaluating the same histopathological parameters, energy dosages, and treatment schedules for both studies would help validate the efficacy of using a 635 nm range wavelength.

In closed wounds, Lyons et al. conducted a study on hairless mice (n = 30) given 6 mm surgical incisions on their backs [12]. A helium-neon laser with a wavelength of 632.8 nm and an energy density of 1.22 J/cm^2^ was used every other day for 300 seconds per treatment during a total of two months for the laser-treated mice [12]. These were compared with experimental controls. The wound tensile strength and collagen concentration of the wound were examined. In comparison to the former study by Gupta et al., an increase in wound tensile strength and collagen accumulation was also observed for the laser-treated mice, however, with a different wavelength and energy density [12]. The difference between open and closed wounds in correlation to the level of wavelength and energy density may explain these similarities.

Open and closed wound healing using low-level-laser therapy has also been investigated in diabetic mouse models. Yu, Naim, and Lanzafame inflicted diabetic mice (n = 40) with two circular 6 mm open wounds on the dorsal thorax of each mouse [13]. The experimental group was treated for 250 seconds daily for a total of four days with an argon dye laser at a wavelength of 630 nm and an energy density of 5 J/cm^2^ [13]. The percentage of wound closure, formation of granulation tissue, collagen deposition, and wound epithelialization were examined. The LLLT stimulated cellular proliferation, enhanced wound healing, and caused the release of growth factors in the laser-treated group [13]. In another open wound study conducted by Tatmatsu-Rocha et al., 20 diabetic and nondiabetic mice were given a 2 × 2 cm wound on the dorsal thorax of each mouse [14]. A superpulsed GaAs diode laser with a 904 nm wavelength and a radiant exposure of 18.288 J/cm^2^ were used on a treatment group daily for 5 consecutive days at 60 secs per treatment [14]. Collagen formation, catalase activity, and nitrite concentration of the wounds were evaluated. There was a more significant increase in the production of collagen and a decrease of oxidative and nitrosative stress in the LLLT diabetic group compared to the control nondiabetic groups [14]. Histologically, the untreated diabetic mice also exhibited fewer collagen fibers than any of the laser-treated groups [14]. Although both studies showed beneficial effects in the treatment groups using LLLT, the treatment parameters and outcome measures differed considerably. The extent in which different treatment methodology can produce comparable results should be further explored.

Only one study using closed surgical incisions in diabetic mice was identified. A study conducted by Stadler et al. involved the use of diabetic mice (n = 20) with two dorsal 1 cm closed incisions [15]. The mice were split evenly into control and laser-treated groups which received a daily treatment of a diode laser with an 830 nm wavelength and energy density of 5 J/cm^2^ for 5 consecutive days, either starting on the day of surgery infliction or three days after [15]. The laser-treated group resulted in an increase in wound tensile strength [15]. More studies regarding closed wounds in diabetic mice are needed to better understand the variables associated with that specific condition. Collectively in mice, these preliminary studies suggest that LLLT produces favorable results in wound treatments using a 635 and 810 nm wavelength at an energy density of 4 J/cm^2^ as having the most potential benefit.

### 2.2. Rat

Following mice, rats are among the most common mammalian species used in research. This makes their function as a wound healing model with LLLT more common compared to other species. The following studies using rats were compared and evaluated (see Table 2). Sardari and Ahrari conducted research on white Wistar rats (n = 32) where two 10 mm parallel open wound incisions were made in the buccal mucosa of each rat with one incision being used as the control [16]. Frequency of treatment ranged from 1 to 3 days. A 632.8 nm HeNe laser with an energy density of 1 J/cm^2^ was used daily for a total of 40 secs. Rats were euthanized on day 5 and histological assessments were performed which examined the inflammatory patterns and cell types of the wounds [16]. There was no significant difference found in any of the categories evaluated [10]. In research conducted by Sobral de Melo Rambo et al., 30 and 500 day old Wistar rats (n = 60) were inflicted with four open wounds measuring 8 mm on the middle of the mid-sagittal plane of each rat [4]. The laser treatment occurred every other day for 67 secs until the day of euthanasia which was performed on days 3, 7, or 14 [4]. A DMC Photon Laser III with a 660 nm wavelength and an energy density of 72 J/cm^2^ was used for the laser-treated groups [4]. The results revealed that there was a decreased inflammatory response, increased angiogenesis, and reepithelialization of the wounds in both the younger and older laser-treated rats at all three endpoints [4]. Both studies evaluated the inflammatory response of the groups, yet the latter study examined additional categories. The different results between the two studies could have been caused by the large difference in energy density levels and the type of laser used. Identifying the effect of a 1 J/cm^2^ energy density level compared to 72 J/cm^2^ may help explain the discrepancies between the two studies.

Yasukawa et al. evaluated closed wounds in Sprague-Dawley rats (n = 55) by inflicting three 10 mm long incisions on the dorsal thoracic, lumbar, and buttock regions [17]. The rats were divided into a total of eleven groups based on two different irradiation settings: 8.5 mW with 2.09J/cm^2^ or 17.0 mW with 4.21 J/cm^2^ [17]. The treatment schedule was conducted either on only the first day, the third day, or the fifth day; daily; or every other day. One of the groups served as a control and received no treatment. Each laser-treated group was irradiated for 15 secs using a HeNe laser based on their corresponding treatment schedules. The power output of 17.0 mW being used at an every other day interval had the most favorable results compared to the other multiple groups which exhibited a lower-quality of wound healing [17]. This group had a significant increase in formation of collagen fibers, prevention of excessive tissue inflammation, and the recovery of tissue continuity [17]. In a closed wound study conducted by Suzuki and Takakuda, Sprague-Dawley rats (n = 60) with 15 mm sutured dorsal thoracic incisions were divided based on duration of treatment and energy densities of 1 J/cm^2^ (92 secs), 5 J/cm^2^ (460 secs), or 10 J/cm^2^ (920 secs) [18]. A 660 nm AlGaInP-type diode laser was used once 24 hours after surgery [18]. Rats were sacrificed on either day 3 or 7 from each group for histological evaluations. Compared to the control and other groups, the laser-treated wounds using an energy density of 5 J/cm^2^ resulted in a significant increase in wound strength, formation of collagen fibers, and reduced number of macrophages surrounding the wound area on day 7 [18]. A main difference between these two studies was the use of the power output (mW) of a laser versus the use of wavelength (nm) to categorize the different treatments conducted. Although they both had similar results, another inconsistency is the intervals at which the laser treatments were conducted. They both evaluated the inflammatory components of the wounds and performed histological evaluations examining the formation of collagen fibers in the treated groups. The relationship between the use of power output, wavelength, and treatment scheduling needs to be explored to identify a trend in the positive effects of LLLT.

Research conducted by Aragao de Melo et al. involved the use of adult albino Wistar rats (n = 40) with a sutured 1-inch incision along the abdomen [19]. A GaAlAs 904 nm wavelength laser with an energy density of 3 J/cm^2^ was used. The irradiation occurred daily for 2 minutes at a total of 7 or 14 days depending on the treatment group [19]. The LLLT resulted in enhanced collagen deposition, an increase in neovascularization, and a change in the inflammatory response on both days [19]. No statistical difference was found between the treatment schedules. Surinchak et al. used rats (n = 75) with 6 cm long incisions along their dorsal midlines closed with stainless steel staples [20]. The rats were divided up into control and laser-treated groups and treated twice a day for either 14 or 28 days at a duration of 3 minutes [20]. A HeNe laser with a wavelength of 632.8 nm and an energy density of 2.2 J/cm^2^ was used [20]. An increase in the breaking strength of the incisions in the early stages of the wound healing process was noted in the laser-treated rats at 14 days, but the wound diminished in strength on day 28 for the other group leading the results to be clinically insignificant [20]. Both of these studies used similar energy densities and treatment intervals, yet they yielded different results as evident in Table 2. Not all the variables between the two studies were the same. The use of two distinct lasers with a major difference in wavelength could explain the inconsistent results. Examining the effects of high and low levels of laser wavelengths can help explain the variability in the results. The interest surrounding the healing ability of low-level lasers in healthy rats and the potential outcomes that it may have, has led to the intriguing task of testing this technique on diabetic rat models.

The altered health status of any animal may lead to different wound healing results when their wounds are subjected to LLLT. Eissa and Salih inflicted diabetic Wistar rats (n = 14) with 2.5±0.2 cm dorsum open wounds and divided them into control and laser-treated groups [21]. A 632.8 nm HeNe laser with a power density of 4.00 mW/cm^2^ was used for treatment five times a week for 4 mins per treatment until full closure of the wounds was observed [21]. The diameter of the wounds was evaluated, and it was concluded that the healing rate in the laser-treated groups was reduced by half compared to the control group, making the use of LLLT the faster option [21]. A study by Kilik et al. involved the use of nondiabetic and diabetic Sprague-Dawley rats (n = 48) containing four open wounds on their dorsum measuring 4 mm in diameter [22]. One wound served as the nonirradiated control while the other three were irradiated daily for no more than six days with a different power density and treatment time of either 1 mW/cm^2^ (83 mins 20 secs), 5 mW/cm^2^ (16 mins 40 secs), or 15 mW/cm^2^ (5 mins 33 secs) [22]. A 635 nm GaAlAs diode laser with an energy density of 5 J/cm^2^ was used for treatments [22]. The 5 mW/cm^2^ and 15 mW/cm^2^ laser-treated wounds experienced an increase in collagen fiber formation, stimulation of neovascularization, and a decrease in inflammatory tissue [22]. Both studies used a similar wavelength and treatment interval, but they evaluated different factors. The former study by Eissa and Salih only evaluated the healing rate of the wounds limiting the findings of the LLLT [21]. The study by Kilik et al. examined more of the histological aspects of the laser-treated wounds leading to multiple beneficial results [22]. The duration of treatment for both studies was also different. The study for Kilik et al. showed that treatment functioned best at 5 mins 33 secs and at 16 mins 40 secs, in comparison with the study by Eissa and Salih, where treatment showed positive results at 4 mins. This indicates that the level of power densities used for these studies could alternatively be a major factor to the results. Evaluating the relationship between power density and treatment schedule can warrant a more conclusive explanation.

Dancakova et al. divided up nondiabetic and diabetic Sprague-Dawley rats (n = 21) into three groups: control, diabetic sham-treated control, and diabetic laser-treated [23]. Each rat was inflicted with a 40 mm long sutured incision and a 4 mm in diameter open wound along their dorsum [23]. The laser-treated group wounds were irradiated for 30 seconds daily for a total of seven days with an 810 nm diode laser and an energy density of 0.9 J/cm^2^ [23]. The laser-treated diabetic group resulted in similar results as the control nondiabetic group. Both showed a higher degree of mature granulation tissue, increased wound tensile strength, and an acceleration of the wound healing process as compared to the sham-treated diabetic rats [23]. In a study by Dadpay et al., adult healthy Wistar rats (n = 18) were split into three groups with one of the groups being induced with type I diabetes [24]. Each rat received two sutured 1.5 cm long incisions with the proximal wound serving as the treatment group and the distal as a control [24]. Treatment occurred daily 6 times a week until day 15 using an 890 nm pulsed infrared diode laser [24]. One group was treated for 30 secs at an energy density of 0.03 J/cm^2^ and two additional groups were treated for 200 secs at an energy density of 0.2 J/cm^2^, with one of these groups containing the diabetes mellitus induced rats [24]. The nondiabetic and diabetic groups treated at an energy density of 0.2 J/cm^2^ showed a more significant increase in wound tensile strength, elastic modulus, and an acceleration of wound healing compared to the 0.03 J/cm^2^ group [24]. Both of the above wound studies in diabetic rats derived similar results. The similarities could be due to the comparable wavelengths and low energy densities used in each study. Overall across these studies, wavelength and treatment scheduling appeared to be the most distinguishing factors that was linked to the beneficial effects of LLLT. Daily laser treatment with a wavelength range of 600 or 800 nm proved to have the most advantageous results.

## 3. Rabbit

Another common small mammal used in laboratory settings is the rabbit. Various studies evaluating the use of LLLT in the wound healing of a rabbit model were identified (see Table 3). In an open wound study by Surinchak et al., two circular holes measuring 16 cm were cut into the left and right dorsal thorax of New Zealand white rabbits (n=34) [20]. Eight rabbits were given treatment every third day for 30 minutes using a 632.8 nm low-level helium-neon laser with an energy density of 1.1 J/cm^2^ [20]. The remaining 26 rabbits were given treatment twice daily for 3 mins at a time until wound closure using the same laser with an energy density of 2.2 J/cm^2^ [20]. At the 80% wound healing mark, the rate of healing and breaking strength of the wounds were measured. The wounds did not show an accelerated effect in healing or breaking strength [20]. In a similar study by Hodjati et al., rabbits (n = 34) were inflicted with a 3 × 3 cm open wound [25]. A diode helium-neon low-intensity laser was used with a wavelength of 808 nm and an energy density of 4 J/cm^2^ [25]. Wound measurements, biopsies, and the grading of epithelialization, inflammation, and fibrosis were evaluated. The LLLT accelerated wound epithelialization [25]. Both open wound studies used a treatment time interval of every three days, yet only Hodjati et al. exhibited positive results. The higher level of wavelength and energy density from Hodjati et al. may be a good indicator of the laser parameters to use for treatment at an interval of every three days, in comparison with Surinchak et al., who used much lower ranges and exhibited insignificant results from the LLLT.

In a closed wound study by Alipanah, Asnaashari, and Anbari, rabbits (n = 16) received a sutured bilateral incision on the buccal gingiva maxilla with the right side being the control and the left the laser-treated [26]. The left side of the maxilla was treated once for one minute following surgery with a pulsed GaAlAs laser with a wavelength of 685 nm and an energy density of 3 J/cm^2^ [26]. The inflammation of the wounds was evaluated based on their intensity using an inflammation score, and a microscopic assessment was made which evaluated the inflammatory cells and rate of inflammation [26]. The laser-treated group had a shortened inflammatory phase and the wound advanced faster into the proliferative and maturation phases of wound healing in comparison to the control group [26].

An open wound study conducted by Hussein et al. evaluated the use of domestic rabbits (n=20) with a 4 × 3 cm wound on the dorsal gluteal region [27]. The rabbits were evenly divided into control and treatment groups. A gallium aluminum arsenide diode laser with a wavelength of 890 nm and a power output of 10 m was used for this study. Treatments were conducted daily for five minutes over a 7-day period [27]. Macroscopic and histological evaluations were performed postoperatively on days 3, 7, and 14. Overall, the laser-treated group exhibited a closed wound with less of a scar formation compared to the control. It also contained a thicker layer of connective tissue and an increase in neutrophils [27].

In research conducted by Braverman et al., New Zealand rabbits (n=72) were inflicted with two 1.5 cm dorsal midline incisions [28]. The rabbits were divided into one control and three laser-treated groups. The experimental groups were treated with either a 632.8 nm HeNe laser at an energy density of 1.65 J/cm^2^, a 904 nm gallium aluminum arsenide (GaAlAs) diode laser at an energy density of 8.25 J/cm^2^, or both [28]. Treatments were performed daily for 11 mins over a period of 21 days. The tensile strength, wound area, and the epidermal thickness of the wounds were evaluated. There was no significant difference in the wound healing rate by measurement of wound area. The wounds treated with the HeNe laser exhibited a slight increase in epidermal growth and all the laser-treated groups showed a significant increase in tensile strength [28].

A similar study by Atabey et al. used rabbits (n=28) with either two 2 × 2 cm open wounds or 3 × 3 cm skin graft wounds bilaterally on the middle flanks [29]. Wounds on the left side were the controls and the ones on the right were treated daily with a 632.8 nm HeNe laser at an energy density of 3.8 J/cm^2^ [29]. Each treatment session was 15 minutes long and the wounds were treated until complete wound contraction was exhibited, which was between 10 and 17 days [29]. The contraction rate and epithelialization of the wounds were evaluated. The results showed no difference in the formation of granulation tissue and wound contraction rate between the laser-treated and control groups [29]. The limited findings in closed and open wound healing using LLLT in rabbits indicate that this area of research must be expanded to obtain the desired laser parameters for this species.

## 4. Canine

Multiple journal articles were identified evaluating LLLT in a canine model (see Table 4). In a study by Kurach et al., adult beagles (n = 10) received two 2 × 2 cm^2^ open wounds on each side of the torso [30]. One wound served as the control, while the other was treated with a dual diode laser at 635 nm and a total energy density of 1.125 J/cm^2^ [30]. The laser treatment occurred three times a week for 32 days with a duration of 300 seconds per treatment [30]. A study conducted by Gammel et al. also involved 10 canines which received a bilateral flank ovariectomy [31]. Each side contained a closed incision and a 15 mm biopsy punch which was left open [31]. One side of the flank served as the control, while the other was treated daily with a low-level laser at 980 nm and an energy density of 5 J/cm^2^ for five consecutive days [31]. Both of these acute wound studies evaluated similar categories including a subjective wound evaluation based on the appearance, fluid color, hydration status, and granulation tissue of the wound; the total wound area, healing time, percentage of contraction/epithelialization, and a histological assessment, which evaluated the amount of collagen formation, inflammatory cell types, and level of necrosis. The laser-treated wounds versus the control groups in both the open and closed wound studies showed no significant differences in any of the categories evaluated.

In a case study, Lucroy, Edwards, and Madewell used LLLT for the closure of a chronic wound that had failed to heal after surgical closure, antibiotic therapy, and bandage application [32]. The veterinarian decided to allow the wound to heal by contraction and epithelialization with the use of an argon-pumped dye laser daily at a wavelength of 630 nm and energy density of 5 J/cm^2^ [32]. The laser treatment occurred for 4 days at a duration of 250 seconds per treatment. Wound surface area and formation of granulation tissue were evaluated. The LLLT resulted in a decrease in wound surface area, a completely healed wound by day 21, and no increased granulation tissue [32]. In comparison to the two former studies, this case study by Lucroy et al. suggests that the condition of the wound and its level of chronicity may affect the results of LLLT, but this is difficult to confirm without a control [32]. This case study strengthens the results evaluated in rats. It demonstrated that LLLT produced more favorable results treating with a daily exposure of a 630 nm wavelength laser, as compared to using a higher wavelength or treating for just three times a week.

In research conducted by Perego et al., ovariectomized dogs (n=7) with a 3 cm sutured abdominal incision were subjected to low-level-laser therapy [33]. Half of the incision was treated with laser while the other half served as the control. An 808 nm gallium aluminum arsenide (GaAlAs) laser with an energy density of 0.9 J/cm^2^ was used on the treated area twice daily at 6 minutes per session for a total of 5 days [33]. From a 0-4 scale, the physical appearance of the wounds was recorded evaluating the color of the wound, pain response, granulation tissue, exudate, and eschar [33]. Although the physical appearance of the treated and nontreated areas was visibly different, including a reduction of exudate on the treated portion, the statistical analysis considered the findings insignificant [33]. This result could have been due to the small sample size of seven dogs and the fact that no histological characteristics were evaluated. Evaluating more parameters would have resulted in a larger margin of error and possibly a different result.

A study conducted by Wardlaw et al. used dogs (n=12) with sutured linear incisions caused by a thoracolumbar hemilaminectomy procedure [34]. The incision was subjected to low-level-laser therapy to evaluate its effectiveness in wound healing. The dogs were split into control and treatment groups. An 850 nm light therapy laser at an energy density of 8 J/cm^2^ was used daily over a period of seven days [34]. The length of the treatment sessions was not provided. The physical appearance of the scars was evaluated on a scale from zero to five on days 1, 3, 5, 7, and 21. Zero was noted as being a fresh incision and five was visible wound contraction and hair growth [34]. The daily application of this laser has been shown to have a significant increase in the macroscopic appearance and healing of the scar [34]. More studies regarding the use of LLLT in canines and other species in refractory wounds will help identify if there is any explanation for the differing results depicted.

## 5. Equine

Only two studies using LLLT for the healing of an open wound were identified in equines (see Table 5). In a study conducted by Jann et al., 8 horses received a single 2.5cm square wound on the mid-metacarpal region of one leg [35]. The horses were randomly split into control and laser-treated groups. A dual diode laser system with a wavelength of 635 nm and an energy density of 5.1 J/cm^2^ was used every other day for five minutes for a total of 80 days [35]. The rate of wound healing and a histological evaluation which consisted of the formation of granulation tissue, ulceration, inflammation, and presence of inflammatory cells were examined. The rate of wound healing was increased for the laser-treated group in comparison to the control group [35]. A study conducted by Petersen, Botes, Olivier, and Guthrie involved 6 crossbred horses each having a 3 × 3 cm open square wound inflicted on the dorsal metacarpophalangeal joints of both legs, with one leg serving as the control and the other as the laser-treated [36]. The treatment consisted of a gallium aluminum arsenide (GaAlAs) laser with a wavelength of 830 nm and an energy density of 2 J/cm^2^ performed every 24 hours for 30 days at a duration of 66 seconds per treatment [36]. The degree of exudation, granulation tissue, pain, wound area, and area of epithelialization were recorded. No significant differences were found in wound contraction or epithelialization between the laser-treated and control groups [36]. Both studies used a different type of laser and combination of wavelength and total energy density. Laser treatment was also given at different time intervals which may have contributed to the different results. No studies evaluating the effects of LLLT in the closed wound healing of equines were found during literature search. More studies using LLLT in open and closed wounds in equine will give more insight into the effectiveness of this modality for this species.

## 6. Conclusion

Researchers must first master the effects of wound LLLT before this type of treatment technique can be incorporated into a larger scale for veterinary clinics or large animal practices. Many other variables besides species must be further examined to determine what effect they have on each study. Regardless of open or closed wounds, there was not an obvious difference between species that indicated ideal laser treatment parameters. Although the results were not the same between species, they were significant in some. Daily laser exposure at a wavelength of a 600 or 800 nm range showed most consistent benefits across the rodent studies examined (refer to Tables 1 and 2). Since the use of rodents in research is immense, data is more readily available compared to the other species examined. In general, there was an uneven representation of open and closed wound studies preventing a feasible conclusion based on wound type. The fact that results differed in the studies suggests that more research is needed on species differences and the effect of LLLT. Other key components that should be considered when evaluating LLLT studies are the health status of the animal, the gender, the differing laser parameters, and the treatment schedule of the laser as those may explain some of the variety noted in these studies. Minimizing the number of factors that can affect a LLLT study will bring about more consistency to the results. More studies in all species are recommended to improve and validate our knowledge about the effects of low-level lasers on wound healing.

## Figures and Tables

**Table 1:** Mouse open and closed wound studies.

Author	Wound Type	Wavelength	Energy Density	Tx Schedule	Outcome
Demidova-Rice et al.	Open	635±15 nm	1, 2, 10, & 50 J/cm^2^	Single exposure	Positive
		632.8 nm	1 & 2 J/cm^2^		(635±15 nm at 2 J/cm^2^)
		670, 720, & 820 (±15 nm)	1 J/cm^2^		(820±15 nm at 1 J/cm^2^)
Gupta, Dai, & Hamblin	Open	635±15, 730, 810, & 980 nm	4 J/cm^2^	Daily for 7 days	Positive (635±15 & 810 nm)
Lyons et al.	Closed	632.8 nm	1.22 J/cm^2^	Every other day for 2 months	Positive
Yu, Naim, & Lanzafame	Open Diabetic	630 nm	5 J/cm^2^	Daily for 4 days	Positive
Tatmatsu-Rocha et al.	Open Diabetic/Non-Diabetic	904 nm	18.288 J/cm^2^	Daily for 5 days	Positive
Stadler et al.	Closed Diabetic	830 nm	5 J/cm^2^	Daily for 5 days	Positive

**Table 2:** Rat open and closed wound studies.

Author	Wound Type	Wavelength	Energy Density	Tx Schedule	Outcome
Sardari & Ahrari	Open	632.8 nm	1 J/cm^2^	(i) Daily (ii) Single Exposure (iii) Every other day (iv) First two days	Statistically Insignificant
Sobral de Melo Rambo et al.	Open	660 nm	72 J/cm^2^	Every other day	Positive
Yasukawa et al.	Closed	8.5 mW 17.0 mW	2.09 J/cm^2^ 4.21 J/cm^2^	(i) Daily (ii) Every other day (iii) Single Exposure	Positive (17.0 mW at 4.21 J/cm^2^, every other day)
Suzuki & Takakuda	Closed	660 nm	1 J/cm^2^ 5 J/cm^2^ 10 J/cm^2^	Single Exposure	Positive (5 J/cm^2^)
Aragao de Melo et al.	Closed	904 nm	3 J/cm^2^	Daily	Positive
Surinchak et al.	Closed	632.8 nm	2.2 J/cm^2^	2x Daily	Statistically Insignificant
Eissa & Salih	Open Diabetic	632.8 nm	4.00 mW/cm^2^	Daily 5x a wk.	Positive
Kilik et al.	Open Diabetic/Non-Diabetic	635 nm	5 J/cm^2^	Daily	Positive
Dancakova et al.	Open/Closed Diabetic/Non-Diabetic	810 nm	0.9 J/cm^2^	Daily	Positive
Dadpay et al.	Closed Diabetic/Non-Diabetic	890 nm	0.03 J/cm^2^ 0.2 J/cm^2^	Daily 6x a wk.	Positive (0.2 J/cm^2^)

**Table 3:** Rabbit open and closed wound studies.

Author	Wound Type	Wavelength	Energy Density	Tx Schedule	Outcome
Surinchak et al.	Open	632.8 nm	1.1 J/cm^2^ 2.2 J/cm^2^	(i) Every 3rd Day (ii) 2x Daily	Statistically Insignificant
Hodjati et al.	Open	808 nm	4 J/cm^2^	Days 0, 3, & 6	Positive
Alipanah et al.	Closed	685 nm	3 J/cm^2^	Single Exposure	Positive
Hussein et.al	Open	890 nm	Unprovided	Daily	Positive
Braverman et al.	Open	632.8nm 904 nm	1.65 J/cm^2^ 8.25 J/cm^2^	Daily	Positive
Atabey et al.	Open & Denuded	632.8 nm	3.8 J/cm^2^	Daily	Positive

**Table 4:** Canine open and closed wound studies.

Author	Wound Type	Wavelength	Energy Density	Tx Schedule	Outcome
Kurach et al.	Open	635 nm	1.125 J/cm^2^	3x a week	No Significant Differences
Gammel et al.	Open/Closed	980 nm	5 J/cm^2^	5 Consecutive Days	No Significant Differences
Lucroy, Edwards, & Madewell	Open	630 nm	5 J/cm^2^	Daily	Positive
Perego et al.	Closed	808 nm	0.9 J/cm^2^	Daily	Statistically Insignificant
Wardlaw et al.	Closed	850 nm	4 J/cm^2^	Daily	Positive

**Table 5:** Equine open wound studies.

Author	Wound Type	Wavelength	Energy Density	Tx Schedule	Outcome
Jann et al.	Open	635 nm	5.1 J/cm^2^	Every Other Day	Positive
Petersen, Botes, Olivier, & Guthrie	Open	830 nm	2 J/cm^2^	Daily	No significant differences

## References

[1] Demidova-Rice T. N., Salomatina E. V., Yaroslavsky A. N., Herman I. M., Hamblin M. R. (2007). Low-level light stimulates excisional wound healing in mice. *Lasers in Surgery and Medicine*.

[2] Mester E., Spiry T., Szende B., Tota J. G. (1971). Effect of laser rays on wound healing. *The American Journal of Surgery*.

[3] Chung H., Dai T., Sharma S. K., Huang Y.-Y., Carroll J. D., Hamblin M. R. (2012). The nuts and bolts of low-level laser (Light) therapy. *Annals of Biomedical Engineering*.

[4] Rambo C. S. O. M., Silva J. A. N., Serra A. J. O. (2014). Comparative analysis of low-level laser therapy (660 nm) on inflammatory biomarker expression during the skin wound-repair process in young and aged rats. *Journal of Lasers in Medical Sciences*.

[5] Velnar T., Bailey T., Smrkolj V. (2009). The wound healing process: an overview of the cellular and molecular mechanisms. *Journal of International Medical Research*.

[6] Walker M. D., Rumpf S., Baxter G. D., Hirst D. G., Lowe A. S. (2000). Effect of low-intensity laser irradiation (660 nm) on a radiation- impaired wound-healing model in murine skin. *Lasers in Surgery and Medicine*.

[7] De Freitas L. F., Hamblin M. R. (2016). Proposed mechanisms of photobiomodulation or low-level light therapy. *IEEE Journal of Selected Topics in Quantum Electronics*.

[8] Farivar S., Malekshahabi T., Shiari R. (2014). Biological effects of low level laser therapy. *Journal of Lasers in Medical Sciences*.

[9] Passarella S., Casamassima E., Molinari S. (1984). Increase of proton electrochemical potential and ATP synthesis in rat liver mitochondria irradiated in vitro by helium-neon laser. *FEBS Letters*.

[10] Basford J. R. (1989). Low-energy laser therapy: Controversies and new research findings. *Lasers in Surgery and Medicine*.

[11] Gupta A., Dai T., Hamblin M. R. (2014). Effect of red and near-infrared wavelengths on low-level laser (light) therapy-induced healing of partial-thickness dermal abrasion in mice. *Lasers in Medical Science*.

[12] Lyons R. F., Abergel R. P., White R. A., Dwyer R. M., Castel J. C., Uitto J. (1987). Biostimulation of wound healing in vivo by a helium-neon laser. *Annals of Plastic Surgery*.

[13] Yu W., Naim J. O., Lanzafame R. J. (1997). Effects of photostimulation on wound healing in diabetic mice. *Lasers in Surgery and Medicine*.

[14] Tatmatsu-Rocha J. C., Ferraresi C., Hamblin M. R. (2016). Low-level laser therapy (904 nm) can increase collagen and reduce oxidative and nitrosative stress in diabetic wounded mouse skin. *Journal of Photochemistry and Photobiology B: Biology*.

[15] Stadler I., Lanzafame R. J., Evans R. (2001). 830-nm irradiation increases the wound tensile strength in a diabetic murine model. *Lasers in Surgery and Medicine*.

[16] Sardari F., Ahrari F. (2016). The effect of low-level helium-neon laser on oral wound healing. *Dental Research Journal*.

[17] Yasukawa A., Ohrui H., Koyama Y., Nagai M., Takakuda K. (2007). The effect of Low reactive-Level Laser Therapy (LLLT) with Helium-Neon laser on operative wound healing in a rat model. *Journal of Veterinary Medical Science*.

[18] Suzuki R., Takakuda K. (2016). Wound healing efficacy of a 660-nm diode laser in a rat incisional wound model. *Lasers in Medical Science*.

[19] de Melo V. A., dos Anjos D. C. S., Albuquerque Júnior R., Melo D. B., Carvalho F. U. R. (2011). Effect of low level laser on sutured wound healing in rats. *Acta Cirurgica Brasileira*.

[20] Surinchak J. S., Alago M. L., Bellamy R. F., Stuck B. E., Belkin M. (1983). Effects of low‐level energy lasers on the healing of full‐thickness skin defects. *Lasers in Surgery and Medicine*.

[21] Eissa M., Salih W. H. M. (2017). The influence of low-intensity He-Ne laser on the wound healing in diabetic rats. *Lasers in Medical Science*.

[22] Kilik R., Lakyova L., Sabo J. (2014). Effect of equal daily doses achieved by different power densities of low-level laser therapy at 635 nm on open skin wound healing in normal and diabetic rats. *BioMed Research International*.

[23] Dancakova L., Vasilenko T., Kovac I. (2014). Low-level laser therapy with 810 nm wavelength improves skin wound healing in rats with streptozotocin-induced diabetes. *Photomedicine and Laser Surgery*.

[24] Dadpay M., Sharifian Z., Bayat M., Bayat M., Dabbagh A. (2012). Effects of pulsed infra-red low level-laser irradiation on open skin wound healing of healthy and streptozotocin-induced diabetic rats by biomechanical evaluation. *Journal of Photochemistry and Photobiology B: Biology*.

[25] Hodjati H., Rakei S., Johari H. G., Geramizedeh B., Sabet B., Zeraatian S. (2014). Low-level laser therapy: an experimental design for wound management: a case-controlled study in rabbit model. *Journal of Cutaneous and Aesthetic Surgery*.

[26] Alipanah Y., Asnaashari M., Anbari F. (2011). The effect of low level laser (GaAlAs) therapy on the post-surgical healing of full thickness wounds in rabbits. *Medical Laser Application*.

[27] Hussein A. J., Alfars A. A., Falih M. A. J., Hassan A.-N. A. (2011). Effects of a low level laser on the acceleration of wound healing in rabbits. *North American Journal of Medical Sciences*.

[28] Braverman B., McCarthy R. J., Ivankovich A. D., Forde D. E., Overfield M., Bapna M. S. (1989). Effect of helium‐neon and infrared laser irradiation on wound healing in rabbits. *Lasers in Surgery and Medicine*.

[29] Atabey A., Karademir S., Atabey N., Barutçu A. (1995). The effects of the helium neon laser on wound healing in rabbits and on human skin fibroblasts in vitro. *European Journal of Plastic Surgery*.

[30] Kurach L. M., Stanley B. J., Gazzola K. M. (2015). The effect of low-level laser therapy on the healing of open wounds in dogs. *Veterinary Surgery*.

[31] Gammel J. E., Biskup J. J., Drum M. G., Newkirk K., Lux C. N. (2018). Effects of low-level laser therapy on the healing of surgically closed incisions and surgically created open wounds in dogs. *Veterinary Surgery*.

[32] Lucroy M. D., Edwards B. F., Madewell B. R. (1999). Low-intensity laser light-induced closure of a chronic wound in a dog. *Veterinary Surgery*.

[33] Perego R., Proverbio D., Spada E., Zuccaro A. (2016). First experience with photobiomodulation (PBM) in post-surgical wound healing in dogs. *Journal of Veterinary Clinical Practice and Petcare*.

[34] Wardlaw J. L., Gazzola K. M., Wagoner A. (2019). Laser therapy for incision healing in 9 dogs. *Frontiers in Veterinary Science*.

[35] Jann H. W., Bartels K., Ritchey J. W., Payton M., Bennett J. M. (2012). Equine wound healing: Influence of low level laser therapy on an equine metacarpal wound healing model. *Photonics and Lasers in Medicine*.

[36] Petersen S. L., Botes C., Olivier A., Guthrie A. J. (1999). The effect of low level laser therapy (LLLT) on wound healing in horses. *Equine Veterinary Journal*.

